# Ascariasis cholecystitis: An unusual cause

**DOI:** 10.4103/0972-9941.45207

**Published:** 2008

**Authors:** Balakrishna Shetty, Prashanth Kumar Shetty, Pritam Sharma

**Affiliations:** Department of General Surgery, K. S. Hegde Medical Academy, Mangalore, India

**Keywords:** Ascariasis, cholecystitis, cholecystectomy

## Abstract

Ascariasis is the most common helminthic infection to infest man. Usually the adult worm lives in the small intestine. Rarely it migrates through the ampulla of vater and enters the common bile duct. We are reporting a case of gall bladder ascariasis causing acute cholecystitis treated by laparoscopic cholecystectomy. Presence of Ascaris lumbricoides in gallbladder is rare entity as it is difficult to reach there due to the narrow and tortuous cystic duct.

## INTRODUCTION

Ascariasis, a human disease caused by roundworm Ascaris lumbricoides. A common disease of the tropics. Parasites can migrate into the biliary tract and create problems such as recurrent pyogenic cholangitis, gall stones, and pancreatitis.[1] Acalculous cholecystitis caused by A. lumbricoides is seen in endemic areas.[1] We are reporting a case of gall bladder (GB) ascariasis, presenting as cholecystitis.

## CASE REPORT

**Figure 1:**
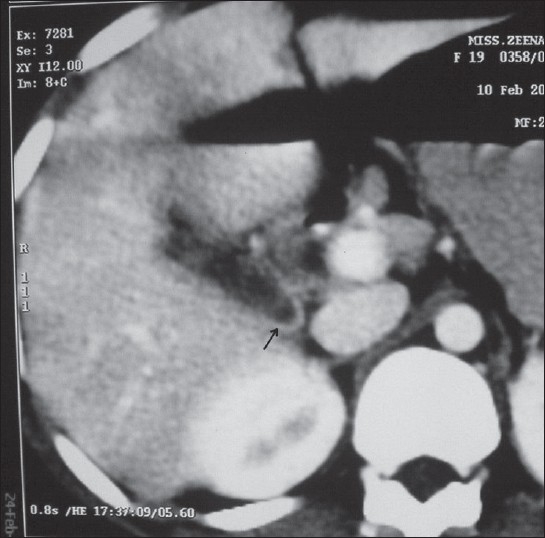
CT scan of hepatobiliary system

**Figure 2:**
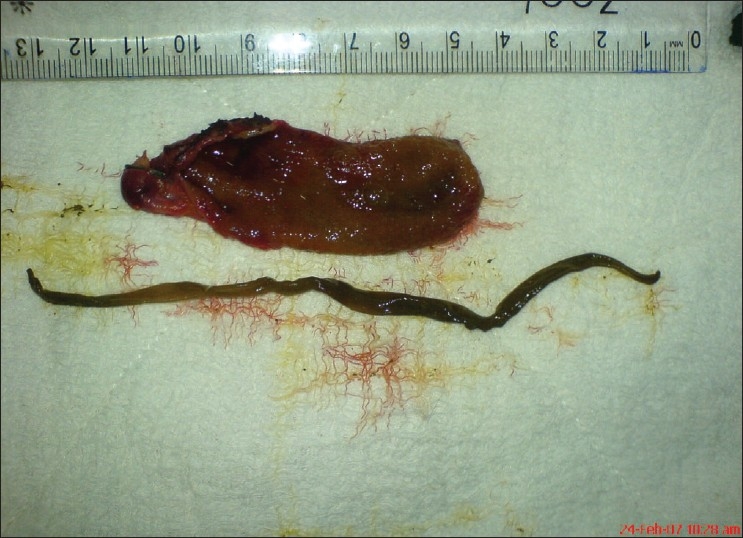
Ascarias lumbricoides

18-year-old girl, of low socio-economic strata, presented with history of right upper abdomen pain of 1-month duration. Aggravation of pain, low-grade fever and non bilious vomiting since 1 day. There was no history of jaundice or of passage of worms in stools. On examination, there was no pallor, no icterus. Abdomen examination revealed tenderness in right hypochondriac region, no palpable mass, no hepatomegaly. Her investigations revealed hemoglobin: 11g%, total count of 12,000/dl, neutrophils: 62%, lymphocytes: 24%, eosinophils: 10%, ESR : 12 mm in first hour. Liver function tests were normal, Alkaline phosphatase was normal (64U/L). Ultrasonography showed a linear, ribbon-like, tubular, non-shadowing structure with an echogenic wall and a less echogenic centre in the GB, suggestive of biliary ascariasis. GB wall thickness was 5 mm, with dilated common bile duct (CBD) (9mm). There was no stone within the GB or CBD. A computerised tomographic (CT) scan [Figure 1] revealed tubular foci (arrow) in the lumen of the GB suggestive of ascariasis, with mild dilatation of CBD at proximal end with smooth tapering at the lower end. No dilatation of intrahepatic biliary radicals. With a diagnosis of acalculous cholecystitis, she was treated with intravenous ceftriaxone and metronidazole. After 3 days of treatment her symptoms subsided. She completed a 7-day course of intravenous antibiotics. An ultrasonography was done at 7 days which showed the worm within the GB. The CBD did not show presence of any worms but it remained minimally dilated measuring 7 mm. She then underwent a laparoscopic cholecystectomy. Specimen of the GB revealed a dead A. lumbricoides measuring 13.5 cm in length [Figure 2]. Histopathology revealed features of eosinophilic cholecystitis. She had an uneventful postoperative period and discharged on the 2^nd^ post operative day with antihelmenthic medications.

## DISCUSSION

Although intestinal and bile duct ascariasis is common, A. lumbricoides in the GB is uncommon, even in endemic areas.[1]

Biliary ascariasis may be complicated or uncomplicated.[2] In uncomplicated biliary ascariasis, the clinical picture merges with that of acute acalculus cholecystitis with low grade fever, upper abdominal colic, tenderness, muscle guarding in right upper quadrant with a gall bladder mass. It maybe complicated by acute cholangitis with fever, right hypochondriac pain, jaundice, tender hepatomegaly, raised bilirubin, alkaline phosphatase, and transaminases. Cholangitis may be suppurative and patient may present with shock.[2]

Diagnosis of biliary ascariasis can be established by hepatobiliary ultrasonography, CT scan. GB may be normal or have thickened wall. Dilatation of biliary duct may be present if worm obstructs the CBD.

Ultrasonography is a highly sensitive and specific method of detection of worms in the biliary tree. It maybe repeated frequently to monitor the movement of worms in the duct.[3]

The established treatments for biliary ascariasis are antihelminthic drug therapy, endoscopic extraction, and surgical extraction.[4,5]

Antihelminthic drug therapy is commonly performed before or after surgery.[4]

In this case, we gave the patient the antihelminthic drug after the laparoscopic cholecystectomy because in our patient the worm was located in the gallbladder, and cases with failed medical treatment have been reported.[5]

Conservative treatment usually fails in the presence of a dead worm, concomitant stones or stricture which prevents the returning of worm to the duodenum, requiring a cholecystectomy or CBD exploration. Laparoscopic[4] or endoscopic procedures (ERCP) are useful in the management of ascariasis in the bile duct.[3]

Our patient presented with ascariasis cholecystitis which was treated with antibiotics.

She was evaluated by ultrasound abdomen and CT scan. After the patient recovered her acute phase, she was re-evaluated with a transabdominal ultrasonography. The worm persisted in the gall bladder with no evidence of worm in CBD. She was then taken up for a laparoscopic cholecystectomy which revealed a dead worm. This patient would have required CBD exploration (open/laparoscopic/endoscopic) if a worm was within the CBD.
